# A Bragg Wavelength-Insensitive Fiber Bragg Grating Ultrasound Sensing System that Uses a Broadband Light and No Optical Filter

**DOI:** 10.3390/s110706954

**Published:** 2011-07-04

**Authors:** Hiroshi Tsuda

**Affiliations:** National Institute of Advanced Industrial Science & Technology, AIST Tsukuba Central 2, Tsukuba, 305-8568, Japan; E-Mail: hiroshi-tsuda@aist.go.jp; Tel.: +81-29-861-9284; Fax: +81-29-861-5881

**Keywords:** fiber Bragg grating sensors, ultrasound, nondestructive testing, acoustic emission, signal processing

## Abstract

An optical filter is incorporated in a conventional ultrasound detection system that uses a fiber Bragg grating (FBG) and broadband light source, to demodulate the FBG sensor signal. A novel ultrasound sensing system that does not require an optical filter is presented herein. Ultrasound could be detected via the application of signal processing techniques, such as signal averaging and frequency filters, to the photodetector output that corresponds to the intensity of the reflected light from a broadband light-illuminated FBG. Ultrasonic sensitivity was observed to be enhanced when an FBG was installed as a resonant sensor. This FBG ultrasound detection system is small and cheap to fabricate because it does not use a demodulating optical filter. The experimental results demonstrate that this system could be applied to ultrasonic damage inspection and acoustic emission measurements. Furthermore, this system was able to detect ultrasound despite the amount of strain or temperature that was applied to the FBG sensor because the ultrasound detection was not sensitive to the Bragg wavelength of the FBG sensor.

## 1. Introduction

Recently, there has been a focus on the development of smart structures that feature self-diagnosing functionalities in their structural integrities. A fiber Bragg grating (FBG) is expected to act as a sensor for smart structures because an FBG is small, lightweight, and immune to electromagnetic interference, and, furthermore, it is capable of multiple functions. An FBG consists of a single-mode optical fiber core that has a periodic variation in its refractive index. This variation causes the reflection of a narrow band of light, and the central wavelength therein is referred to as the Bragg wavelength. The Bragg wavelength varies based on the strain and temperature that are applied to the FBG. The strain and temperature sensitivities of an FBG with a Bragg wavelength of 1,550 nm are 14 pm/K and 1.2 pm/μɛ, respectively [1].

Ultrasound can be detected by an FBG because it causes a minute shift in the Bragg wavelength. Previously proposed FBG ultrasound detection systems can be classified as either one of two types based on the light source that is employed. One type uses a broadband light source, wherein an optical filter is included to demodulate the FBG sensor signal. The spectrum of the optical filter must overlap with the reflective spectrum of the FBG sensor to detect ultrasound. The other type of light source for ultrasound detection involves a laser-demodulation system, wherein a laser is tuned to a wavelength where the slope of the FBG reflective spectrum is steep. The details regarding the principles of ultrasound detection via both systems have been described in a previous study [2].

In most studies, ultrasonic FBG sensors have been embedded in or glued to the structure that was to be monitored [3–6]. The direct attachment of FBGs enables sensitive ultrasound detection; however, the FBG reflective spectrum shifts based on the strain and temperature that are applied to the structure, and the reflective spectrum can deviate from the wavelength range where ultrasound can be detected when the structure is subjected to significant changes in strain or temperature. Using a tunable laser or a tunable optical filter, ultrasound sensitivity can be maintained by controlling the wavelength of the laser or the filter spectrum in accordance with the shift in the FBG reflective spectrum; however, demodulation control cannot follow rapid strain and temperature changes. As such, demodulation is unlikely to continuously detect acoustic emission (AE) because AE accompanies sudden strain change due to material failure.

Lee and the author have proposed a strain-insensitive FBG ultrasonic sensor in which an FBG-inscribed optical fiber without the grating section is attached to a monitored material [7]. Ultrasound propagating in the monitored material travels along the optical fiber via the point of contact. Next, the ultrasound reaches the FBG and impinges upon it. The author has demonstrated continuous AE measurement during a CFRP tank pressure test by using a laser-demodulation system in combination with a strain-insensitive FBG sensor [8]; however, the ultrasonic sensitivity of a strain-insensitive FBG sensor wanes under varying temperature conditions because temperature influences the Bragg wavelength of the FBG sensor.

Lee *et al*. have also developed a Bragg wavelength-insensitive FBG ultrasonic detection system that uses a broadband light source [9]. In this system, two Fabry-Perot filters with free spectral ranges (FSRs) that correspond to the full bandwidth of the FBG sensor are used as demodulators. This system is capable of detecting ultrasound regardless of the Bragg wavelength of the FBG sensor when the transmission peak wavelengths of the two demodulators are separated from one another by a quarter of the FSR; however, there are several drawbacks to this system. For example, a thermoregulator must be installed to control the wavelength of the Fabry-Perot filter spectrum, and this addition increases the size, weight, and power supply requirements of the system. Furthermore, the ultrasound sensitivity of this system periodically depends on the Bragg wavelength of the FBG sensor.

A compact ultrasound detection system is required for structural health monitoring in aerospace applications. In this study, an FBG ultrasound detection system that features a simple configuration with a broadband light source and does not require an optical filter was developed. The results indicate that this system is capable of detecting ultrasound regardless of the Bragg wavelength of the FBG sensor. Moreover, the ultrasound detection sensitivity can be enhanced via the use of a resonant FBG sensor.

## 2. Ultrasound Detection Using the Developed FBG Sensing System

**Figure 1.**
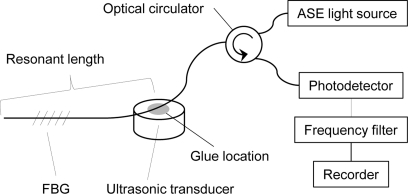
Experimental setup for FBG ultrasound detection without an optical filter for demodulation.

To the author’s knowledge, all previously reported FBG ultrasound detection systems in which a broadband light was used as the light source have utilized optical filters for FBG sensor signal demodulation [2,6,9–11]; however, the experiment discussed herein demonstrates that a system that uses a broadband light source is capable of detecting ultrasound without optical demodulation using optical filters. The experimental setup for the system is depicted in Figure 1. An FBG was illuminated by a broadband light via an optical circulator, and the light that was reflected from the FBG was transmitted into a photodetector wherein the reflected light intensity was converted into a voltage signal.

**Figure 2.**
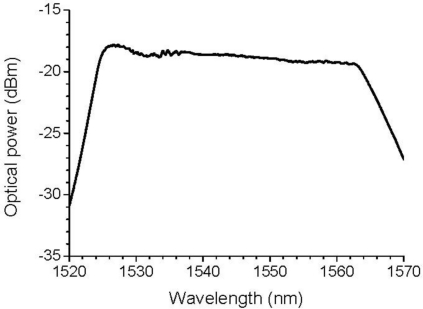
Optical power distribution of the employed broadband light source.

The optical power distribution of the broadband light source that was employed in this experiment is depicted in Figure 2. The output of the photodetector, which was an electric signal, was recorded after going through an electric frequency filter. Part of an optical fiber, in which a 10-mm-long FBG with a Bragg wavelength of 1,550 nm was inscribed, was bonded to an ultrasonic transducer with a central frequency of 250 kHz (V150, Panametrics). A pulse of 375 V was emitted to the transducer as an excitation signal and ultrasound was generated from the transducer. In this study, the length of the optical fiber between its free end and the point that was bonded to the ultrasound transducer is referred to as the resonant length, as depicted in Figure 1. The photodetector output, which corresponded to the FBG sensor response to ultrasound, was recorded using different resonant lengths, including 0 mm, 50 mm, and 291 mm. An FBG sensor with a resonant length of 0 mm means that the FBG was directly bonded to the ultrasonic transducer.

**Figure 3.**
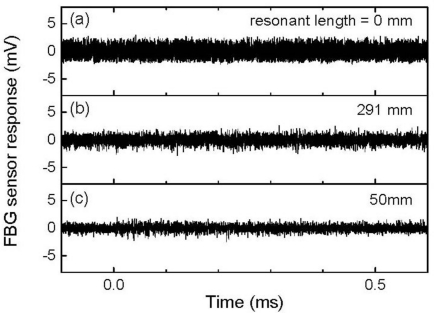
Responses to individual ultrasonic pulses that were detected by FBG sensors with resonant lengths of (**a**) 0mm, (**b**) 291 mm, and (**c**) 50 mm.

**Figure 4.**
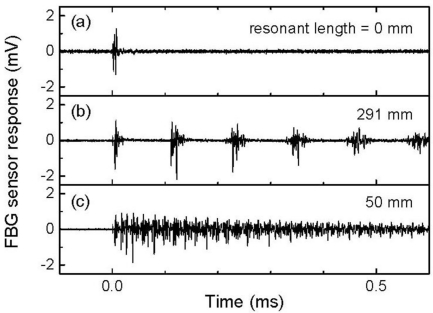
The 512-time-averaged ultrasonic responses of FBG sensors with resonant lengths of (**a**) 0 mm, (**b**) 291 mm, and (**c**) 50 mm.

Figure 3 depicts 500-kHz-low-pass-filtered FBG sensor responses as a function of different resonant lengths to individual ultrasonic pulses, wherein there are no observable ultrasonic responses in these signals. Figure 4 depicts 512-time-averaged FBG sensor responses that were acquired using the same frequency filter conditions. Using the ultrasound detection system without an optical filter demodulation, a well-defined ultrasonic response could be obtained by averaging the signals of the FBG sensor output. The FBG sensor with a resonant length of 0 mm demonstrated a single ultrasonic response although that with a resonant length of 291 mm responded repeatedly every 0.11 ms. This repeated response corresponded to the round-trip ultrasound propagation along the resonant length of the optical fiber. The ultrasonic response continuously appeared in the FBG sensor with a resonant length of 50 mm due to the shorter required time for round-trip ultrasonic propagation.

**Figure 5.**
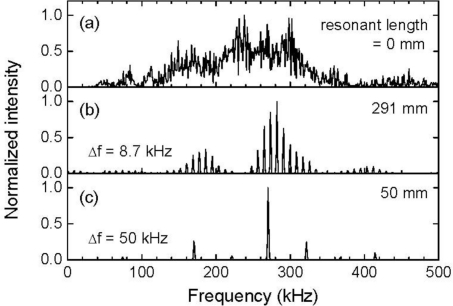
Frequency characteristics of the FBG sensor responses depicted in Figure 4.

Figure 5 depicts the frequency characteristics of the FBG sensor signals depicted in Figure 4. The response of the FBG sensor with a resonant length of 0 mm featured broadband characteristics in which the central frequency of the ultrasonic transducer (250 kHz) was centrally covered. On the other hand, the FBG sensors with resonant lengths of 291 mm and 50 mm demonstrated resonant characteristics with frequency intervals of 8.7 kHz and 50 kHz, respectively.

The FBG sensor of a cantilever beam configuration shows resonant characteristics, which have been reported in a previous paper [12]. The interval of resonant frequencies, Δf_r_, was determined via Equation (1):

(1) $$\Delta f_{r}=\frac{v}{2L}$$

where v and L denote the velocity of the longitudinal-mode wave that propagates along the optical fiber and the resonant length, respectively. The velocity of the longitudinal-mode wave along the optical fiber was reported to be 4,860 m/s [8]. The intervals of the resonant frequencies of the FBG sensors with resonant lengths of 291 mm and 50 mm calculated from Equation (1) were 8.4 kHz and 49 kHz, respectively. These estimated frequency intervals agreed well with the intervals that were experimentally obtained.

**Figure 6.**
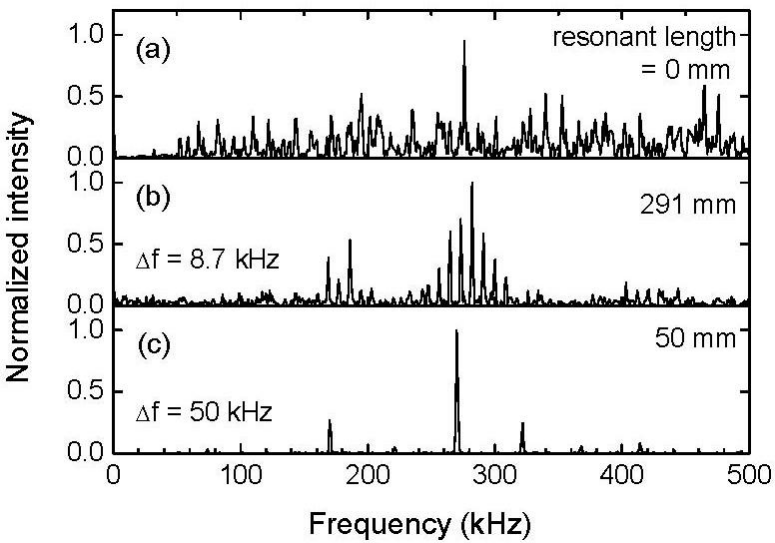
Frequency characteristics of the FBG sensor responses depicted in Figure 3.

Figure 6 depicts the frequency characteristics of the non-averaged FBG sensor signals that are depicted in Figure 3. Although the averaged response of the FBG sensor with a resonant length of 0 mm possessed high intensity frequency components around 250 kHz which was the central frequency of the ultrasonic transducer, both response signals with a resonant length of 0 mm shown in Figure 5(a) and Figure 6(a) exhibited essentially broadband characteristics. The other two responses with the same resonant lengths exhibited resonant frequency intervals that were identical to those obtained from the averaged FBG sensor signals.

In Figure 6, it is worth noting the frequency characteristics of the FBG sensor signal that had a resonant length of 50 mm. Frequencies with high component intensities appeared at 320 kHz or lower. Based on this result, the ultrasound response can be extracted from the noisy FBG sensor signal through an appropriate low-pass filter process from the FBG sensor signal.

**Figure 7.**
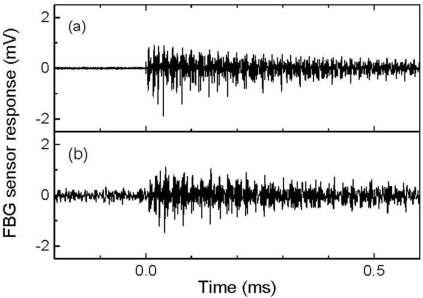
The ultrasonic responses of an FBG sensor with a resonant length of 50 mm. (**a**) The 512-time-averaged response that was acquired through a low-pass filter at a cut-off frequency of 500 kHz, (**b**) The non-averaged response that was acquired through a low-pass filter at a cut-off frequency of 350 kHz.

Figure 7(a,b) depict the 512-time-averaged FBG sensor signal that was acquired through a 500-kHz low-pass filter and the non-averaged signal that was obtained through a 350-kHz low-pass filter, respectively. An evident ultrasonic response began at the same time even though the non-averaged signal exhibited noticeable noise that appeared before the response. These experiments demonstrate that ultrasound can be detected by averaging or filtering the FBG sensor output without a demodulation using an optical filter.

Although the optical power distribution at 1,550 nm, which corresponds to the Bragg wavelength of the employed FBG sensor, seems flat as shown in Figure 2, it varies slightly depending on the wavelength. The ultrasound detection of the system discussed herein must utilize a slight wavelength distribution of optical power that is emitted from the light source. Thus, the ultrasound detection must not be sensitive to the Bragg wavelength because the optical power that is emitted from the broadband light source depends on variations in the wavelength. Similar ultrasound detection tests were performed using FBGs with different Bragg wavelengths, and the results of these experiments demonstrate that ultrasound is capable of being detected despite the Bragg wavelengths of the FBG sensors.

## 3. The Application of the Developed Ultrasound Detection System to Ultrasonic Damage Inspection

**Figure 8.**
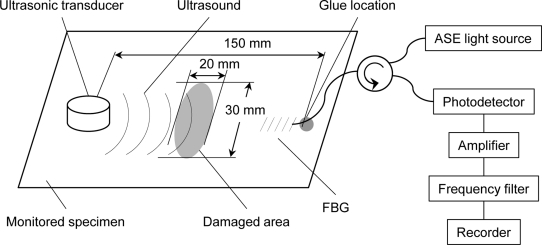
Experimental setup for ultrasonic damage inspection.

The applicability of the developed ultrasound detection system to ultrasonic damage inspection was verified via the experimental setup depicted in Figure 8. The monitored specimen involved a cross-ply CFRP plate with 30 mm × 20 mm of damage. The damage was induced by a dropping ball impact. Splitting and delamination could be seen in the damaged area. Part of a 10-mm-long, FBG-inscribed optical fiber was glued to the monitored specimen, and the resonant length was 52 mm. Ultrasound was excited under the same conditions as those used in the experiment described in the previous section. The span between the ultrasonic transducer and the glued point of the FBG sensor was 150 mm. The FBG sensor responses to ultrasound that was propagated through intact and damaged areas were recorded through a 46-dB gain amplifier and a 1-MHz low-pass filter.

**Figure 9.**
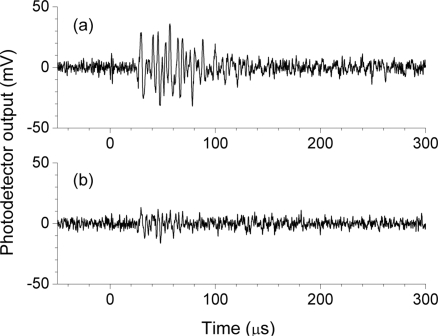
FBG sensor responses to ultrasound (**a**) that was propagated through an intact area and (**b**) propagated through a damaged area.

The 512-time-averaged responses to ultrasound that was propagated through intact and damaged areas are depicted in Figure 9(a,b), respectively. The intensity of the response to the ultrasound that was propagated through the damaged area markedly decreased.

**Figure 10.**
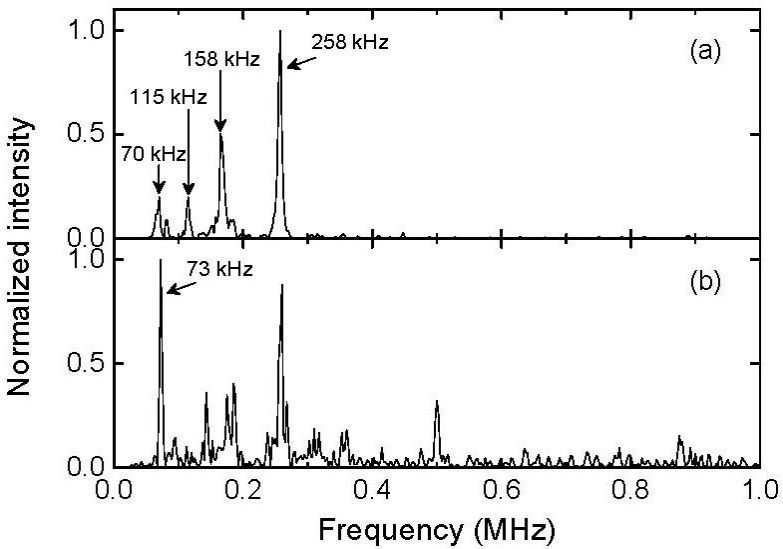
Frequency characteristics of the FBG sensor responses shown in Figure 9.

The frequency characteristics of these response signals are depicted in Figure 10(a,b). The resonant frequency of an FBG sensor with a resonant length L was determined via Equation (2) [12]:

(2) $$f_{n}=\frac{(2n+1)v}{4L},n=0,1,2,\cdots$$

The resonant frequencies of the employed FBG sensor were calculated to be 23, 70, 117, 164, 210, and 257 kHz in order of increasing n in Equation (2). The response to the ultrasound that was propagated through the intact area exhibited the highest component intensity at 258 kHz. This frequency corresponds to the resonant frequency that is closest to the central frequency of a 250 kHz ultrasonic transducer. The response exhibited high component intensities at 70, 115, and 158 kHz, which also corresponded to the resonant frequencies of the FBG sensor. In contrast, the response to the ultrasound that was propagated through the damaged area peaked at a lower frequency of 73 kHz, even though the component intensity at 258 kHz was high. Furthermore, this response exhibited noticeable high component intensities at frequencies of greater than 300 kHz, whereas the response to the ultrasound that was propagated through the intact area exhibited insignificant component intensities. Ultrasound attenuation and scattering in the damaged area must have caused these differences in the response amplitudes and frequency characteristics. The experimental results demonstrate that the developed system is capable of being utilized for ultrasonic damage inspection.

## 4. The Detection of a Quasi-AE Using the Developed Ultrasound Detection System

A system that is capable of detecting AEs must have ultrasonic sensitivity capable of detecting individual ultrasonic pulses that are propagating through a material because AEs are sudden events that are induced by microscopic material failures. The following experiments were performed to validate the AE detection capability of the ultrasound detection system. Ultrasound, which was excited under the same conditions as those used in the previous experiments, was propagated through a 1-mm-thick aluminum plate. As described in Section 2, an FBG with a fully glued grating can be used as a broadband ultrasonic sensor. On the other hand, an FBG with an optical fiber that lacks the glued grating section can be used as a resonant ultrasonic sensor. An individual ultrasound pulse detection test was performed by using both broadband and resonant FBG ultrasonic sensors. The glued location of the FBG sensor was placed 150 mm away from the ultrasonic transducer that was used in this study.

**Figure 11.**
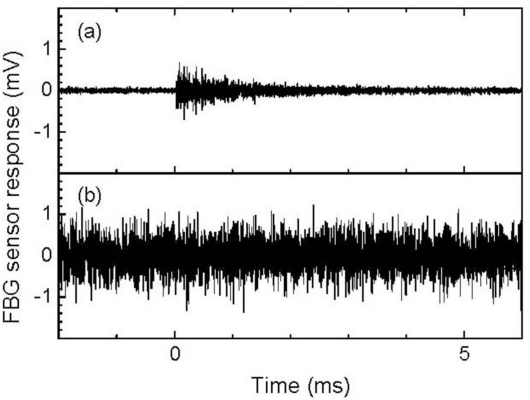
Responses to ultrasound that was propagated through an aluminum plate. These responses were detected via a broadband FBG sensor and acquired through a low-pass filter at a cut-off frequency of 500 kHz. (**a**) The 512-time-averaged response signal and (**b**) the non-averaged response signal.

The 512-time-averaged and non-averaged broadband FBG sensor signals are depicted in Figure 11(a,b), respectively. Both signals were 500-kHz-low-pass-filtered during the data acquisition. The averaged signal exhibited a noticeable ultrasound response, whereas the non-averaged response was indistinguishable from noise.

**Figure 12.**
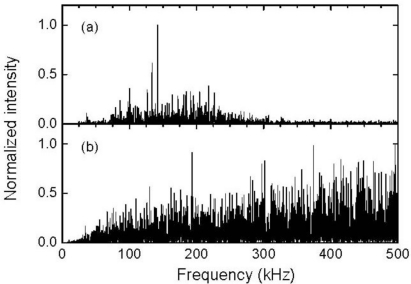
Frequency characteristics of the ultrasonic responses shown in Figure 11.

Figure 12(a,b) depict the frequency characteristics of the broadband FBG sensor signals. The averaged signal exhibited broadband frequency characteristics that ranged from 30 to 300 kHz and exhibited higher intensities from approximately 130 to 140 kHz. On the other hand, the non-averaged signal exhibited a high intensity over the entire range except for the low frequency range, which was less than 20 kHz. Because the noise exhibited a wide frequency range, it is unlikely that an ultrasound response can be extracted from the non-averaged broadband FBG sensor signal using a frequency filter process.

**Figure 13.**
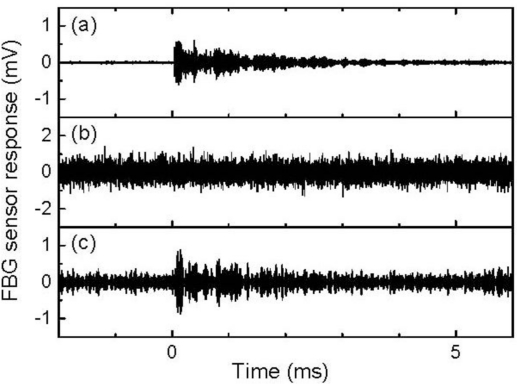
Responses to ultrasound that was propagated through an aluminum plate. These responses were detected via a resonant FBG sensor with a resonant length of 22 mm. (**a**) The 512-time-averaged response through a 500 kHz low-pass filter, (**b**) the non-averaged response through a 500 kHz low-pass filter, and (**c**) the non-averaged response through a 170-kHz band-pass filter.

The 512-time-averaged and non-averaged signals of a resonant FBG sensor with a resonant length of 22 mm are depicted in Figure 13(a,b), respectively. Both responses were 500-kHz low-pass filtered. In comparison to the broadband FBG sensor signal that is depicted in Figure 11(a), the averaged resonant FBG sensor signal exhibited a well-defined ultrasonic response; however, the non-averaged signal exhibited a noisy response that was similar to that detected by the broadband FBG sensor, as depicted in Figure 11(b).

**Figure 14.**
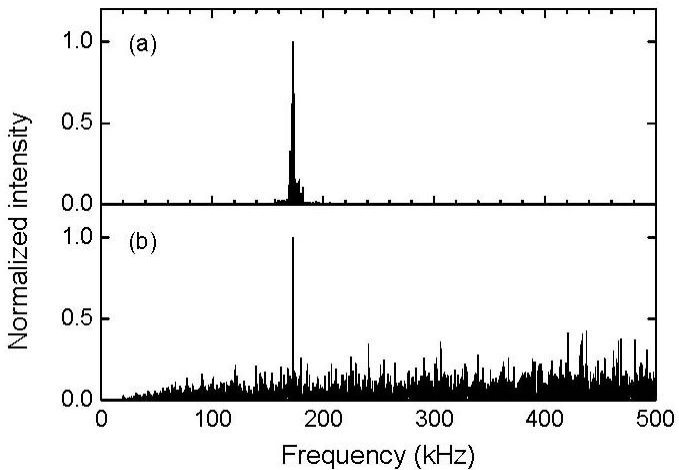
Frequency characteristics of the responses shown in Figure 13(a,b).

Figure 14(a,b) depict the frequency characteristics of the resonant FBG sensor signals. The averaged resonant FBG sensor signal exhibited a high intensity frequency component in the vicinity of 170 kHz. Using Equation (2), the resonant frequencies of the FBG sensor with a resonant length of 22 mm were sequentially calculated to be 55, 166, and 276 kHz from the zero-order. Therefore, the ultrasound was detected by this resonant FBG sensor as a response at the first-order resonant frequency. The non-averaged FBG sensor signal also exhibited a highest intensity at 173 kHz despite the somewhat high intensity of noise that appeared in a wide frequency range. It can be inferred from the localized frequency characteristics of the resonant FBG sensor response that an individual ultrasonic pulse is capable of being detected through a band-pass filter with a center frequency that has the same value as the first-order resonant frequency of the sensor. Figure 13(c) depicts the 170-kHz-band-pass-filtered, and non-averaged FBG sensor signal. The band-pass-filtered signal exhibited an ultrasonic response with amplitude greater than twice the noise level that appeared before the response.

According to the previous study regarding the AE detection of a CFRP filament-wound tank, most of the detected AEs had frequencies ranges of less than 200 kHz [13]. Therefore, the FBG ultrasound detection system developed in this study would be capable of detecting most AEs of CFRP structures in combination with a resonant FBG sensor and an appropriate band-pass filter process.

## 4. Conclusions

A novel FBG ultrasound detection system that uses a broadband light source and no optical filter was developed. The ultrasound detection of this system utilizes wavelength dependence on the optical power that is emitted from a broadband light source. Based on these results, the following conclusions can be made:

1. The FBG functioned as a broadband ultrasonic sensor when the FBG was fully glued to a monitored material. In contrast, the FBG worked as a resonant ultrasonic sensor when part of an FBG-inscribed optical fiber that lacked a grating section was glued to form a cantilever structure. The resonant frequency of a resonant FBG sensor was controlled by changing the resonant length.
2. Ultrasound was detected by the signal averaging of a broadband FBG sensor response. Using a resonant FBG sensor, ultrasound was detected not only through the signal averaging of an FBG sensor response, but also by the band-pass filtering or low-pass filtering of an FBG sensor signal.
3. An impact-damaged CFRP plate was ultrasonically inspected using a resonant FBG sensor. The averaged FBG sensor response to ultrasound that had been propagated through a damaged area could easily be distinguished from ultrasound that had been propagated through an intact area in terms of response amplitude and frequency characteristics.
4. An individual ultrasound pulse that was propagated through an aluminum plate was detected by using a resonant FBG sensor through a narrow band-pass filter with a center frequency that had been set to the resonant frequency of the FBG sensor. AEs are capable of being detected via a resonant FBG sensor through a narrow band-pass filter.
